# Triphlorethol-A from *Ecklonia cava* Up-Regulates the Oxidant Sensitive 8-Oxoguanine DNA Glycosylase 1

**DOI:** 10.3390/md12115357

**Published:** 2014-10-28

**Authors:** Ki Cheon Kim, In Kyung Lee, Kyoung Ah Kang, Mei Jing Piao, Min Ju Ryu, Jeong Mi Kim, Nam Ho Lee, Jin Won Hyun

**Affiliations:** 1 School of Medicine, Jeju National University, Jeju 690-756, Korea; E-Mails: svv771@hotmail.com (K.C.K.); legna07@naver.com (K.A.K.); meijing0219@hotmail.com (M.J.P.); 2 Radiation Effect Research Team, Radiation Health Research Institute, Korea Hydro & Nuclear Power Co., LTD., Seoul 135-881, Korea; E-Mail: inkyeong@korea.ac.kr; 3 Food and Nutrition, Duksung Women’s University, Seoul 132-714, Korea; E-Mail: rmj5924@naver.com; 4 Department of Chemistry, College of Natural Sciences, Jeju National University, Jeju 690-756, Korea; E-Mails: jmkin1263@jejunu.ac.kr (J.M.K.); namho@jejunu.ac.kr (N.H.L.)

**Keywords:** antioxidant response elements, 8-oxoguanine, Nrf2, OGG1, Triphlorethol-A

## Abstract

This study investigated the protective mechanisms of triphlorethol-A, isolated from *Ecklonia cava*, against oxidative stress-induced DNA base damage, especially 8-oxoguanine (8-oxoG), in Chinese hamster lung fibroblast V79-4 cells. 8-Oxoguanine DNA glycosylase-1 (OGG1) plays an important role in the removal of 8-oxoG during the cellular response to DNA base damage. Triphlorethol-A significantly decreased the levels of 8-oxoG induced by H_2_O_2_, and this correlated with increases in OGG1 mRNA and OGG1 protein levels. Furthermore, siOGG1-transfected cell attenuated the protective effect of triphlorethol-A against H_2_O_2_ treatment. Nuclear factor erythroid 2–related factor 2 (Nrf2) is a transcription factor for OGG1, and Nrf2 combines with small Maf proteins in the nucleus to bind to antioxidant response elements (ARE) in the upstream promoter region of the OGG1 gene. Triphlorethol-A restored the expression of nuclear Nrf2, small Maf protein, and the Nrf2-Maf complex, all of which were reduced by oxidative stress. Furthermore, triphlorethol-A increased Nrf2 binding to ARE sequences and the resulting OGG1 promoter activity, both of which were also reduced by oxidative stress. The levels of the phosphorylated forms of Akt kinase, downstream of phosphatidylinositol 3-kinase (PI3K), and Erk, which are regulators of OGG1, were sharply decreased by oxidative stress, but these decreases were prevented by triphlorethol-A. Specific PI3K, Akt, and Erk inhibitors abolished the cytoprotective effects of triphlorethol-A, suggesting that OGG1 induction by triphlorethol-A involves the PI3K/Akt and Erk pathways. Taken together, these data indicate that by activating the DNA repair system, triphlorethol-A exerts protective effects against DNA base damage induced by oxidative stress.

## 1. Introduction

Single bases within a DNA strand can be chemically damaged by deamination, oxidation, or alkylation through a variety of mechanisms [1]. Among the various types of DNA base damage caused by oxidative stress, 8-oxoguanine (8-oxoG) forms abundantly and easily [2,3]. 8-OxoG is formed by reaction of guanine with reactive oxygen species (ROS), and can induce DNA mutation or alteration. Ultimately, such changes can trigger apoptosis and diverse diseases, such as atherosclerosis, diabetes, Parkinson’s disease, and various cancers [4,5,6,7,8]. Single-base lesions such as 8-oxoG are repaired by the base excision repair (BER) system [9], which recognizes and removes damaged and irrelevant bases [10]. A component of the BER system, 8-oxoguanine DNA glycosylase 1 (OGG1), repairs 8-oxoG via the BER pathway by cleaving the glycosidic bond of the 8-oxoG lesion and causing a strand break in the DNA backbone [11,12]. The OGG1 promoter region contains nuclear factor erythroid 2-related factor 2 (Nrf2)-binding sites, called antioxidant response elements (ARE) [13,14]. The transcription factor Nrf2 is essential for ARE-mediated induction of genes encoding phase-II detoxification and oxidative stress-response enzymes [15]. Nrf2 binds to the ARE with high affinity only as a heterodimer with a small Maf protein, suggesting that the Nrf2/small Maf complex activates gene expression directly through the ARE [16]. The small Maf proteins, Maf F, Maf G, and Maf K, contain leucine-zipper (Zip) domains that are required for homodimer or heterodimer complex formation with other Zip transcription factors [17]. To function as transcription factor, Nrf2 must dissociate from Keap1, which degrades Nrf2 by targeting it for ubiquitination, and translocate to the nucleus [15]. The phosphorylation of Nrf2 is the primary signal causing its dissociation from Keap1. The phosphoinositide 3-kinase (PI3K)/Akt pathway may also play an important role in the activation of Nrf2 [18,19]. In addition, Erk phosphorylation activates Nrf2 and induces its translocation to the nucleus [20].

Triphlorethol-A is a phlorotannin compound derived from the brown alga *Ecklonia cava*. Our recent study showed that triphlorethol-A increases heme oxygenase-1 activity by elevating the transcriptional activity of Nrf2 [21], and also ameliorates the effects of formaldehyde on non-homologous end joining and BER capacity [22]. Furthermore, this compound enhances the activities of the antioxidant system and inhibits cellular damage against ultraviolet B rays, thereby protecting human keratinocytes against ultraviolet B radiation [23]. In light of these observations, our current study focused on the ability of triphlorethol-A to protect cells against H_2_O_2_-induced DNA base damage and investigated the molecular mechanisms underlying this protective effect.

## 2. Results

### 2.1. Triphlorethol-A Suppresses H_2_O_2_-induced 8-oxoG Formation

**Figure 1:**
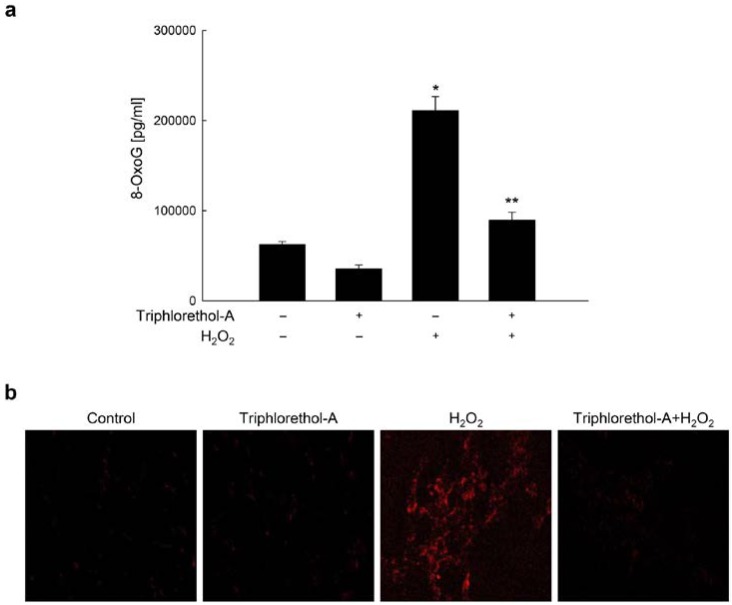
Triphlorethol-A suppresses 8-oxoG generated by H_2_O_2_ treatment. (**a**) Cells were treated with 30 μM triphlorethol-A for 1 h, and then incubated with 1 mM H_2_O_2_ for an additional 24 h. The amount of 8-oxoG in DNA was determined using the Bioxytech 8-OHdG-ELISA kit. * Significantly different from control cells (*p* < 0.05) and ** significantly different from H_2_O_2_-treated cells (*p* < 0.05); (**b**) The binding of avidin-TRITC, which reflects 8-oxoG levels, was visualized with a fluorescence microscope.

We previously reported that optimal dose of triphlorethol-A which protected V79-4 cells against oxidative stress was 30 μM [24]. Therefore, 30 μM of triphlorethol-A was used in these experiments. 8-OxoG is one of the critical forms of ROS-induced oxidative base lesions in DNA, and so has been widely used as a biomarker for oxidative stress and carcinogenesis [25]. We analyzed the 8-oxoG levels in DNA by ELISA assay using specific antibodies against 8-ohdG (a nucleoside of 8-oxoG). Levels of 8-oxoG were significantly higher in H_2_O_2_-treated cells than in control cells; however, triphlorethol-A treatment decreased the levels of 8-oxoG detected in H_2_O_2_-treated cells (Figure 1A). We also estimated the amount of 8-oxoG in a fluorescence-based binding assay using an avidin-conjugated TRITC reagent [26]. The fluorescence intensity generated by 8-oxoG was elevated in H_2_O_2_-treated cells; however, cells treated with H_2_O_2_ and triphlorethol-A exhibited a significantly lower fluorescence intensity (Figure 1B). These results suggest that triphlorethol-A decreases 8-oxoG levels in H_2_O_2_-treated cells.

### 2.2. Triphlorethol-A Reverses the Suppression of OGG1 mRNA and Protein Expression by H_2_O_2_ Treatment

**Figure 2:**
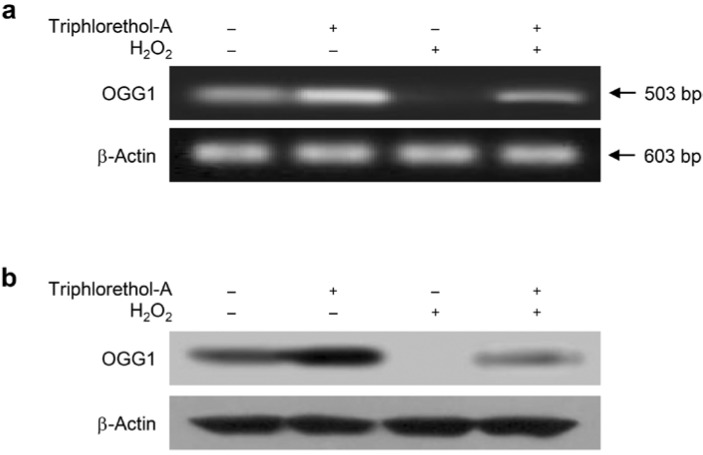
Triphlorethol-A induces OGG1 mRNA and OGG1 protein expression. (**a**) Cells were treated with 30 μM triphlorethol-A for 1 h, and then incubated with 1 mM H_2_O_2_ for an additional 24 h. OGG1 mRNA levels were detected by RT-PCR analysis; (**b**) OGG1 protein levels were detected by Western-blot analysis.

**Figure 3:**
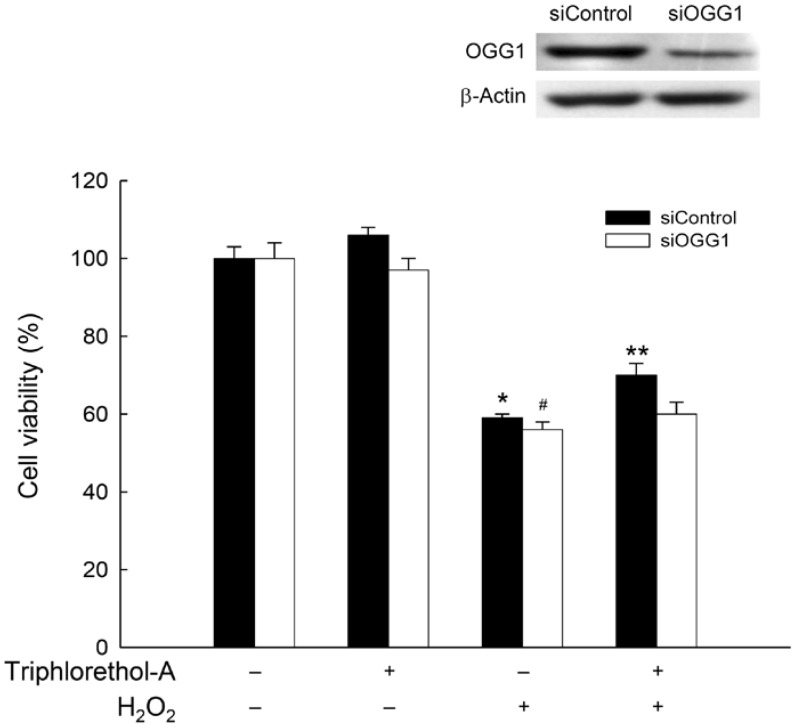
Down-regulation of OGG1 attenuates the cyto-protective effect of triphlorethol-A against H_2_O_2_-treated cells. The siRNA-transfected cells were treated with 30 μM triphlorethol-A for 1 h, and then incubated with 1 mM H_2_O_2_. After 24 h, cell viability was assessed by the MTT assay. * Significantly different from siControl cells (*p <* 0.05); ** significantly different from H_2_O_2_-treated siControl cells (*p <* 0.05), and ^#^ significantly different from siOGG1-transfected cells (*p <* 0.05).

OGG1, a component of the BER system initiated by oxidation of DNA bases, is the primary enzyme responsible for the excision of the 8-oxoG lesion, a mutagenic base byproduct that occurs as a result of exposure to ROS [2]. To determine transcription levels of the OGG1 gene, we measured OGG1 mRNA expression using RT-PCR. Levels of OGG1 mRNA were lower in H_2_O_2_-treated cells than in control cells; however, triphlorethol-A treatment restored the levels of OGG1 mRNA in H_2_O_2_-treated cells to control levels (Figure 2A). Additionally, triphlorethol-A pretreatment significantly increased the levels of OGG1 protein in H_2_O_2_-treated cells compared with those in cells treated with H_2_O_2_ alone (Figure 2B). These data indicate that triphlorethol-A can reverse the reduction in OGG1 transcription and OGG1 protein expression by H_2_O_2_ treatment. Furthermore, cell viability was significantly increased in H_2_O_2_-treated cells treated with triphlorethol-A, compared to H_2_O_2_-treated cells (Figure 3). However, this cyto-protective effect of triphlorethol-A was attenuated in siOGG1-transfected cells, suggesting that OGG1 might involve in cell survival via DNA repair (Figure 3).

### 2.3. Triphlorethol-A Blocks Inhibition of OGG1 Transcription by H_2_O_2_ Treatment

**Figure 4:**
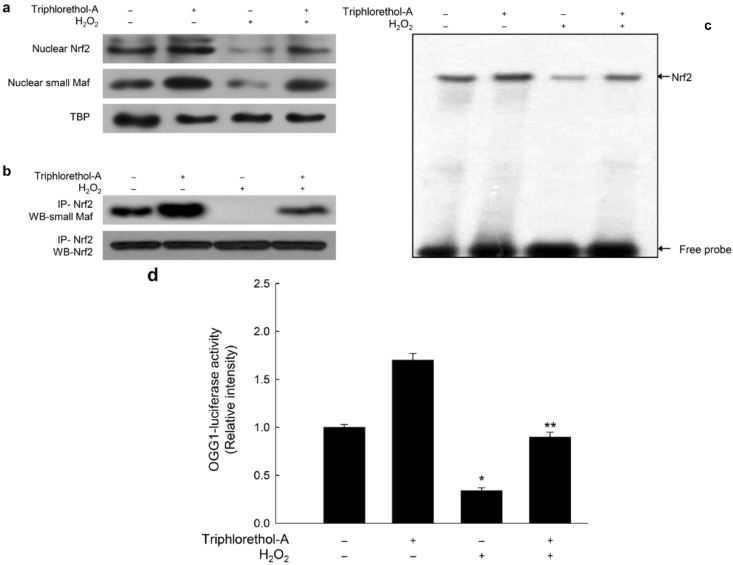
Triphlorethol-A induces expression of Nrf2 and small Maf protein, Nrf2-ARE binding and OGG1 promoter activity. (**a**) Cells were treated with 30 μM triphlorethol-A for 1 h, and then incubated with 1 mM H_2_O_2_ for an additional 12 h. Nuclear extracts were electrophoresed, and nuclear Nrf2, small Maf proteins, and TATA box–binding protein (TBP) were detected using specific antibodies; (**b**) Nuclear extracts were immunoprecipitated with an anti-Nrf2 antibody and subjected to Western blotting using antibodies against small Maf protein (upper) or Nrf2 (lower); (**c**) EMSA was performed with probes containing the ARE sequence from the OGG1 regulatory region. Nuclear extracts were incubated with the probes, and the protein-DNA complexes and free probes were resolved by electrophoresis; (**d**) After overnight transfection with the OGG1 promoter luciferase vector, cells were treated with triphlorethol-A for 1 h and then incubated with 1 mM H_2_O_2_ for a further 12 h. Cells were lysed and cell lysates were mixed with a luciferase substrate. Luciferase activity was measured with a luminometer. * Significantly different from control cells (*p <* 0.05), and ** significantly different from H_2_O_2_-treated cells (*p <* 0.05).

The OGG1 promoter region contains transcription factor binding sites (ARE sequences) for Nrf2 [13,27]. Nrf2 binds to the ARE with a small Maf protein, and activates gene expression directly through the ARE [16]. To analyze the levels of Nrf2 and small Maf proteins in the nucleus, we extracted nuclear proteins and measured the expression of each protein by Western blotting. H_2_O_2_ treatment decreased the nuclear levels of Nrf2 and small Maf proteins; however, triphlorethol-A treatment restored these proteins to control levels (Figure 4A). To determine whether Nrf2 was bound to small Maf proteins in the nucleus, nuclear lysates were immunoprecipitated with an anti-Nrf2 antibody and subjected to Western blotting using an antibody against small Maf proteins. H_2_O_2_ treatment decreased the binding of Nrf2 to small Maf proteins, and triphlorethol-A treatment restored this binding (Figure 4B). Moreover, triphlorethol-A treatment prevented the suppression of Nrf2 binding to the ARE sequence following H_2_O_2_ treatment (Figure 4C). To measure the OGG1 promoter activity, we employed a luciferase reporter vector driven by the promoter region of OGG1. H_2_O_2_ treatment decreased the transcriptional activity of the OGG1 promoter; however, triphlorethol-A treatment restored the promoter activity to control levels (Figure 4D). Thus, in cells treated with H_2_O_2_, triphlorethol-A restored OGG1 expression via up-regulation of Nrf2 and activity of the OGG1 promoter. 

**Figure 5:**
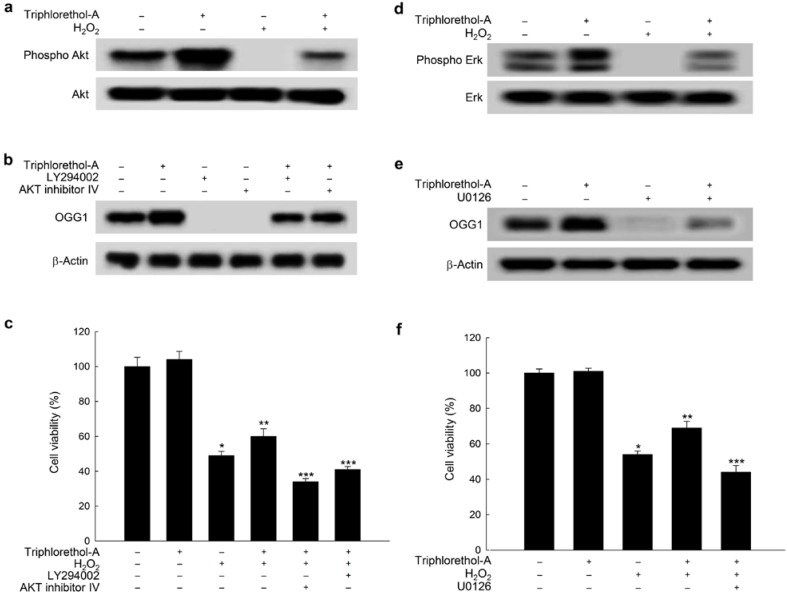
Triphlorethol-A induces OGG1 expression via signaling pathways and improves cell survival. (**a**) Cells were treated with 30 μM triphlorethol-A for 1 h, and then incubated with 1 mM H_2_O_2_ for a further 12 h. Cell lysates were electrophoresed, and Akt and its phosphorylated form were detected using specific antibodies; (**b**) Cells were pretreated with 50 μM LY294002 or 1 μM Akt inhibitor IV for 1 h, and then treated with 30 µM triphlorethol-A for a further 24 h. OGG1 was detected by Western blotting using an anti-OGG1 antibody; (**c**) Cells were pretreated with 50 μM LY294002 or 1 μM Akt inhibitor IV for 1 h, then treated with 30 μM triphlorethol-A for 1 h, and then incubated with 1 mM of H_2_O_2_ for a further 24 h. Cell viability was assessed by the MTT assay. * Significantly different from control cells (*p <* 0.05), ** significantly different from H_2_O_2_-treated cells (*p <* 0.05), and *** significantly different from H_2_O_2_-treated cells also treated with triphlorethol-A (*p <* 0.05); (**d**) Cells were treated with 30 μM triphlorethol-A for 1 h, and were then treated with 1 mM H_2_O_2_ for a further 12 h. Phosphorylated Erk expression was detected by Western blotting; (**e**) Cells were pretreated with 1 μM U0126 for 1 h, and then treated with 30 µM triphlorethol-A for a further 24 h. OGG1 expression was detected by Western blotting; (**f**) Cells were pretreated with 1 μM U0126 for 1 h, then treated with 30 μM triphlorethol-A for 1 h, and then incubated with 1 mM H_2_O_2_ for a further 24 h. Cell viability was assessed by the MTT assay. * Significantly different from control cells (*p <* 0.05), ** significantly different from H_2_O_2_-treated cells (*p <* 0.05), and *** significantly different from H_2_O_2_-treated cells also treated with triphlorethol-A (*p <* 0.05).

### 2.4. Triphlorethol-A Induces OGG1 Expression via the PI3K/Akt and Erk Pathways

The PI3K/Akt pathway is a major signaling process involved in cell survival during oxidative stress. Recently, it was reported that the promoter region contains Nrf2-binding sites, and that the PI3K/Akt pathway is involved in the up-regulation of OGG1 [14]. Akt activity, which correlates with Akt phosphorylation, was decreased upon H_2_O_2_ treatment, but triphlorethol-A treatment restored Akt activation to control levels (Figure 5A). Treatment with LY294002 (a PI3K inhibitor) or Akt inhibitor IV attenuated the ability of triphlorethol-A treatment to restore OGG1 expression (Figure 5B). Likewise, cell viability was inhibited by H_2_O_2_ treatment, but triphlorethol-A treatment partially restored viability to control levels; however, the restoration of cell viability by triphlorethol-A was blocked by treatment with PI3K and Akt inhibitors (Figure 5C). Phosphorylation of Nrf2 by Erk at conserved sites is required for release of Nrf2 from Keap1, and Erk phosphorylation is involved in Nrf2 nuclear translocation; in addition, direct phosphorylation of Nrf2 by Erk is required for Nrf2/DNA binding and subsequent transactivation [20]. Furthermore, under redox stress several effectors translocate Nrf2 from the cytoplasm to the nucleus in response to Erk-mediated Nrf2 phosphorylation at Ser40, and nuclear Nrf2 induces the transcription of the OGG1 gene [13,28]. Triphlorethol-A pretreatment of H_2_O_2_-treated cells restored the levels of phosphorylated Erk to control levels, whereas the level in cells treated with H_2_O_2_ alone was markedly reduced (Figure 5D). Treatment with U0126, an Erk inhibitor, suppressed the restoration of OGG1 expression by triphlorethol-A treatment in H_2_O_2_-treated cells (Figure 5E). Furthermore, U0126 abolished the protective effect of triphlorethol-A against H_2_O_2_-induced cell death (Figure 5F). These results indicate that the PI3K/Akt and Erk pathways partially regulate Nrf2 transcriptional activity and OGG1 expression, and thereby affect cell viability.

## 3. Experimental Section

### 3.1. Reagents

The plasmid containing the OGG1 promoter luciferase construct was a generous gift from Professor Ho Jin You (Chosun University, Gwangju, Korea). Avidin-conjugated tetramethylrhodamine isothiocyanate (TRITC), [3-(4,5-dimethylthiazol-2-yl)-2,5-diphenyltetrazolium] bromide (MTT), and an antibody against OGG1 were purchased from Sigma-Aldrich Corporation (St. Louis, MO, USA). Antibodies against Nrf2, small Maf, phospho-Akt, Akt, phospho-Erk, Erk, and β-actin were purchased from Santa Cruz Biotechnology (Santa Cruz, CA, USA). TATA box binding protein (TBP) antibody was purchased from Abcam (Cambridge, MA, USA). LY294002, Akt inhibitor IV and U0126 were purchased from Calbiochem (San Diego, CA, USA).

### 3.2. Isolation of Triphlorethol-A

The *E*. *cava* was dried in the shade and cut into small pieces. The dried sample (2.0 kg) was extracted with 80% aqueous methanol at room temperature for 24 h. The combined solution was filtered, and the filtrate was concentrated under reduced pressure to afford the extract (259 g). The obtained extract was suspended on water, and partitioned into *n*-hexane (6.3 g) and ethyl acetate (30.8 g)-soluble fractions. The ethyl acetate fraction was subjected to column chromatography over celite by eluting successively with *n*-hexane, methylene chloride, diethyl ether and methanol to give four fractions. The third fraction (10.0 g) eluted with diethyl ether was purified by column chromatography with eluent of chloroform/methanol (2/1) to give 21 fractions (fr. 1-21). The fraction 14 (323.8 mg) was identified to be triphlorethol-A as determined by spectroscopic method including the analysis of magnetic resonance spectroscopy (NMR) spectra, and confirmed by the comparison of the data to the literature values [29].

### 3.3. Cell Culture

Chinese hamster lung fibroblasts (V79-4) were obtained from the American Type Culture Collection (Rockville, MD, USA) and cultured in Dulbecco’s modified Eagle’s medium containing 10% heat-inactivated fetal calf serum, streptomycin (100 μg/mL), and penicillin (100 Units/mL). Cells were maintained at 37 °C in an incubator with a humidified atmosphere of 5% CO_2_. The cell doubling time of V79-4 is approximately 12 h, and cells were used for this study at 60%–70% confluence.

### 3.4. Detection of 8-oxoG

Cellular DNA was isolated using DNAzol reagent (Life Technologies, Grand Island, NY, USA) and quantified using a spectrophotometer. The amount of 8-hydroxy-2-deoxyguanosine (8-ohdG; a nucleoside of 8-oxoG) in the DNA was determined using the Bioxytech 8-OHdG ELISA kit from OXIS Health Products (Portland, OR, USA) according to the manufacturer’s instructions. The detected 8-ohdG level was considered to represent the 8-oxoG level. The amount of 8-ohdG was also estimated using a fluorescence-based binding assay [26]. Avidin binds with high specificity to both 8-oxoG and 8-ohdG. Cells were fixed and permeabilized with ice-cold methanol for 15 min and incubated with avidin-conjugated TRITC (fluorescent dye) for 1 h at room temperature. 8-OxoG was visualized using a fluorescence microscope. 

### 3.5. Reverse Transcriptase-Polymerase Chain Reaction (RT-PCR)

Total RNA was isolated from cells using easy-BLUE^™^ (iNtRON Biotechnology, Kyounggi, Korea). PCR conditions for OGG1 and the housekeeping gene β-actin were as follows: 94 °C for 2 min; 35 cycles of 94 °C for 20 s, 58 °C for 30 s, and 72 °C for 1 min; and 72 °C for 5 min. The primer pairs (Bioneer Corporation, Daejeon, Korea) were as follows: mouse OGG1, sense 5′-GCAGAGCCCTGCTCACTGGA-3′ and antisense 5′-CGAGGATGGCTTTGGCACTG-3′; mouse β-actin, sense 5′-GTGGGCCGCCCTAGGCACCAGG-3′ and antisense 5′-GGAGGAAGAGGATGCGGCAGTG-3′. Amplified products were resolved by 1% agarose gel electrophoresis, stained with ethidium bromide, and photographed under ultraviolet light.

### 3.6. Extraction of Total Cellular Proteins and a Nucleus Protein Fraction

Cells were seeded in a plate at a concentration of 1 × 10^5^ cells/mL. To extract total cellular proteins, cells were lysed on ice for 30 min in 150 μL of PRO-PREP™ (iNtRON Biotechnology, Kyounggi, Korea) and centrifuged at 13,000 rpm for 30 min. To extract nuclear proteins, cells were lysed using the Subcellular Protein Fractionation kit (Thermo Scientific, Milwaukee, WI, USA) according to the manufacturer’s instructions.

### 3.7. Western Blotting

Cell or nuclear lysates were collected, and protein concentrations were determined using the Bradford reagent. Aliquots of the lysates (40 μg of protein) were boiled for 5 min and electrophoresed on 10% SDS-polyacrylamide gels. Gels were transferred onto nitrocellulose membranes (Bio-Rad, Hercules, CA, USA). Membranes were then incubated with the indicated primary antibodies, and further incubated with secondary immunoglobulin G–horseradish peroxidase conjugates. Protein bands were visualized by developing the blots using an Enhanced Chemiluminescence (ECL) Western blotting detection kit (Amersham, Buckinghamshire, UK) and exposing the ECL-treated membranes to X-ray film.

### 3.8. Immunoprecipitation 

Cell lysates were mixed with Nrf2 antibody and shaken at 4 °C overnight. Next, 30 μL of protein G–agarose beads were added to the lysates, and the mixture was shaken for 2 h at 4 °C. The lysates were centrifuged at 6000 rpm for 5 min, and the protein G–agarose beads were collected. Collected beads were incubated with elution buffer for 30 min on ice, and then centrifuged at 13,000 rpm for 5 min. Supernatants were collected, and protein concentrations were determined using the Bradford reagent.

### 3.9. Electrophoretic Mobility Shift Assay (EMSA)

Oligonucleotides containing the Nrf2-binding domain (ARE sequence) were labeled with [γ-^32^P] ATP using T4 polynucleotide kinase, and the labeled oligonucleotides were used as probes. The probes (50,000 cpm) were incubated with the nuclear extracts at 4 °C for 30 min in a final volume of 20 μL containing 12.5% glycerol, 12.5 mM HEPES (pH 7.9), 4 mM Tris-HCl (pH 7.9), 60 mM KCl, 1 mM EDTA, 1 mM DTT, and 1 μg of poly(dI–dC). Binding products were resolved on 5% polyacrylamide gels, and the bands were visualized by autoradiography.

### 3.10. Transient Transfection and OGG1-Promoter Luciferase Assay

Cells were transiently transfected with a reporter plasmid harboring the OGG1 promoter using the transfection reagent DOTAP (Roche, Mannheim, Germany) according to the manufacturer’s instructions. After overnight transfection, cells were treated with triphlorethol-A for 1 h, and 1 mM H_2_O_2_ was then added to the medium. After 24 h, the cells were lysed with reporter lysis buffer (Promega, Madison, WI, USA). The lysate supernatant was then mixed with the luciferase assay reagent, and the mixture was placed in a luminometer to measure the light produced.

### 3.11. Transient Transfection of Small Interfering RNA (siRNA)

Cells were seeded to 60 mm cell culture dish at a density of 1.5 × 10^5^ cells/mL and allowed to grow 70% confluency. Cells were transfected with 10 nM of siControl (Santa Cruz, CA, USA) and 50 nM of siRNA against OGG1 (Bioneer, Daejeon, Korea) by using Lipofectamine™ 2000 (Invitrogen Inc., Carlsbad, CA, USA) based on the manufacturer’s instructions. Twenty-four hours after transfection, cells were treated with triphlorethol-A for 1 h, and 1 mM H_2_O_2_ was then added to the medium. After 24 h, the cell viability was examined.

### 3.12. Cell Viability

Cells were seeded in a 96-well plate at a concentration of 1.0 × 10^4^ cells/well and pretreated with 50 μM of LY294002, 1 μM of Akt inhibitor IV, or 1 μM of U0126 for 1 h, followed by treatment with 30 μM of triphlorethol-A for 1 h and treatment with 1 mM H_2_O_2_ for 24 h. MTT (50 µL of a 2 mg/mL stock solution) was then added to each well to yield a total reaction volume of 200 μL. After incubating for 4 h, the plate was centrifuged at 1500 rpm for 5 min, and the supernatants were aspirated. The formazan crystals in each well were dissolved in 150 μL dimethylsulfoxide, and the A_540_ was read on a scanning multi-well spectrophotometer [30].

### 3.13. Statistical Analysis

All values are represented as the mean ± standard error (SE). The results were subjected to an analysis of variance (ANOVA) using Tukey’s test for analysis of differences. Statistical significance was set at *p* < 0.05.

## 4. Discussion and Conclusions

Oxidative stress reflects an imbalance between the systemic manifestation of ROS and the ability of a biological system to readily detoxify the reactive intermediates or to repair the resulting damage. Disturbances in the normal redox state of cells can cause toxic effects through the production of peroxides and free radicals that damage all components of the cell, including proteins, lipids, and DNA [31,32]. In particular, extreme oxidative stress can induce DNA base modifications such as 8-oxoG, which can generate DNA mutations and ultimately trigger apoptosis and various diseases [33,34]. During DNA synthesis, 8-oxoG forms a mismatched base pair with adenine, giving rise to a G:C-to-T:A transversion mutation, potentially altering gene function and resulting in cell death [35,36]. The modified base is removed by DNA glycosylase, and the lesion site subsequently undergoes repair processing by BER systems [37]. OGG1 acts as an 8-oxoG glycosylase and endonuclease during the BER process under conditions of oxidative stress [10,12]. The OGG1 gene is transcriptionally activated by Nrf2, which binds to ARE sequences in the OGG1 promoter [13,14]. Under non-stressed conditions, Nrf2 is anchored in the cytoplasm by binding to Keap1, which facilitates the ubiquitination and subsequent proteolysis of Nrf2 [15,38]. Thus, Keap1 negatively regulates Nrf2 both by enhancing its rate of proteasomal degradation and by altering its subcellular distribution. Upon activation, Nrf2 dissociates from Keap1, translocates into the nucleus, and heterodimerizes with other basic leucine-Zip transcription factors such as small Maf proteins, thereby increasing the transcription of ARE-driven genes [17]. Several mechanisms have been proposed for Nrf2–Keap1 dissociation and regulation of Nrf2 signaling [16]. Severe oxidative stress conditions induce cells to undergo cell death, which exposes surrounding tissues to the vicissitudes of the inflammatory immune response. Cell death may abolish various cellular signaling pathways, including anti-oxidative processes, such as the Nrf2 signaling pathway [39]. Our results presented here suggest that triphlorethol-A can suppress oxidative stresses and thereby protect cells against noxious stimuli, such as H_2_O_2_, formaldehyde, and ionizing radiation [21,22,24,40,41]. Upon exposure to various stress inducers, Nrf2 is released from Keap1 and translocates into the nucleus [42,43]. Nrf2-Keap1 dissociation and Nrf2 signaling are initiated by the phosphorylation of Nrf2 at serine 40 and specific threonine residues, as well as by the modification of cysteine residues in Keap1. Phosphorylation of Nrf2 is performed by various kinases, including mitogen-activated protein kinases, protein kinase C, PI3K/Akt, casein kinase-2, and PKR-like endoplasmic reticulum kinase [44]. In this study, we observed that inhibition of the Erk and PI3K/Akt pathways reduced the up-regulation of OGG1 expression by treatment with triphlorethol-A. Furthermore, these inhibitors suppressed the protective effects of triphlorethol-A against H_2_O_2_ treatment. Triphlorethol-A increased OGG1 expression via the Nrf2 signaling pathway, and thereby protected cells against the extreme oxidative stress induced by H_2_O_2_ treatment. Nrf2 signaling activation, via phosphorylation by Erk and Akt, was also induced by triphlorethol-A. Taken together, these data demonstrate that triphlorethol-A activates the BER system in response to oxidative stress, and thereby prevents DNA base modifications, mutations, and cell death induced by oxidative stress.
